# The null hypothesis significance test in health sciences research (1995-2006): statistical analysis and interpretation

**DOI:** 10.1186/1471-2288-10-44

**Published:** 2010-05-19

**Authors:** Luis Carlos Silva-Ayçaguer, Patricio Suárez-Gil, Ana Fernández-Somoano

**Affiliations:** 1 Centro Nacional de Investigación de Ciencias Médicas, La Habana, Cuba; 2 Unidad de Investigación. Hospital de Cabueñes, Servicio de Salud del Principado de Asturias (SESPA), Gijón, Spain; 3 CIBER Epidemiología y Salud Pública (CIBERESP), Spain and Departamento de Medicina, Unidad de Epidemiología Molecular del Instituto Universitario de Oncología, Universidad de Oviedo, Spain

## Abstract

**Background:**

The null hypothesis significance test (NHST) is the most frequently used statistical method, although its inferential validity has been widely criticized since its introduction. In 1988, the *International Committee of Medical Journal Editors *(ICMJE) warned against sole reliance on NHST to substantiate study conclusions and suggested supplementary use of confidence intervals (CI). Our objective was to evaluate the extent and quality in the use of NHST and CI, both in English and Spanish language biomedical publications between 1995 and 2006, taking into account the *International Committee of Medical Journal Editors *recommendations, with particular focus on the accuracy of the interpretation of statistical significance and the validity of conclusions.

**Methods:**

Original articles published in three English and three Spanish biomedical journals in three fields (General Medicine, Clinical Specialties and Epidemiology - Public Health) were considered for this study. Papers published in 1995-1996, 2000-2001, and 2005-2006 were selected through a systematic sampling method. After excluding the purely descriptive and theoretical articles, analytic studies were evaluated for their use of NHST with P-values and/or CI for interpretation of statistical "significance" and "relevance" in study conclusions.

**Results:**

Among 1,043 original papers, 874 were selected for detailed review. The exclusive use of P-values was less frequent in English language publications as well as in Public Health journals; overall such use decreased from 41% in 1995-1996 to 21% in 2005-2006. While the use of CI increased over time, the "significance fallacy" (to equate statistical and substantive significance) appeared very often, mainly in journals devoted to clinical specialties (81%). In papers originally written in English and Spanish, 15% and 10%, respectively, mentioned statistical significance in their conclusions.

**Conclusions:**

Overall, results of our review show some improvements in statistical management of statistical results, but further efforts by scholars and journal editors are clearly required to move the communication toward ICMJE advices, especially in the clinical setting, which seems to be imperative among publications in Spanish.

## Background

The null hypothesis statistical testing (NHST) has been the most widely used statistical approach in health research over the past 80 years. Its origins dates back to 1279 [1] although it was in the second decade of the twentieth century when the statistician Ronald Fisher formally introduced the concept of "null hypothesis" *H*_0 _- which, generally speaking, establishes that certain parameters do not differ from each other. He was the inventor of the "P-value" through which it could be assessed [2]. Fisher's P-value is defined as a conditional probability calculated using the results of a study. Specifically, the P-value is the probability of obtaining a result at least as extreme as the one that was actually observed, assuming that the null hypothesis is true. The Fisherian *significance testing theory *considered the p-value as an index to measure the strength of evidence against the null hypothesis in a single experiment. The father of NHST never endorsed, however, the inflexible application of the ultimately subjective threshold levels almost universally adopted later on (although the introduction of the 0.05 has his paternity also).

A few years later, Jerzy Neyman and Egon Pearson considered the Fisherian approach inefficient, and in 1928 they published an article [3] that would provide the theoretical basis of what they called *hypothesis statistical testing*. The Neyman-Pearson approach is based on the notion that one out of two choices has to be taken: accept the null hypothesis taking the information as a reference based on the information provided, or reject it in favor of an alternative one. Thus, one can incur one of two types of errors: a *Type I *error, if the null hypothesis is rejected when it is actually true, and a *Type II *error, if the null hypothesis is accepted when it is actually false. They established a rule to optimize the decision process, using the p-value introduced by Fisher, by setting the maximum frequency of errors that would be admissible.

The null hypothesis statistical testing, as applied today, is a hybrid coming from the amalgamation of the two methods [4]. As a matter of fact, some 15 years later, both procedures were combined to give rise to the nowadays widespread use of an inferential tool that would satisfy none of the statisticians involved in the original controversy. The present method essentially goes as follows: given a null hypothesis, an estimate of the parameter (or parameters) is obtained and used to create statistics whose distribution, under *H*_0_, is known. With these data the P-value is computed. Finally, the null hypothesis is rejected when the obtained P-value is smaller than a certain comparative threshold (usually 0.05) and it is not rejected if P is larger than the threshold.

The first reservations about the validity of the method began to appear around 1940, when some statisticians censured the logical roots and practical convenience of Fisher's P-value [5]. Significance tests and P-values have repeatedly drawn the attention and criticism of many authors over the past 70 years, who have kept questioning its epistemological legitimacy as well as its practical value. What remains in spite of these criticisms is the lasting legacy of researchers' unwillingness to eradicate or reform these methods.

Although there are very comprehensive works on the topic [6], we list below some of the criticisms most universally accepted by specialists.

• The P-values are used as a tool to make decisions in favor of or against a hypothesis. What really may be relevant, however, is to get an effect size estimate (often the difference between two values) rather than rendering dichotomous true/false verdicts [7-11].

• The P-value is a conditional probability of the data, provided that some assumptions are met, but what really interests the investigator is the inverse probability: what degree of validity can be attributed to each of several competing hypotheses, once that certain data have been observed [12].

• The two elements that affect the results, namely the sample size and the magnitude of the effect, are inextricably linked in the value of p and we can always get a lower P-value by increasing the sample size. Thus, the conclusions depend on a factor completely unrelated to the reality studied (i.e. the available resources, which in turn determine the sample size) [13,14].

• Those who defend the NHST often assert the objective nature of that test, but the process is actually far from being so. NHST does not ensure objectivity. This is reflected in the fact that we generally operate with thresholds that are ultimately no more than conventions, such as 0.01 or 0.05. What is more, for many years their use has unequivocally demonstrated the inherent subjectivity that goes with the concept of P, regardless of how it will be used later [15-17].

• In practice, the NHST is limited to a binary response sorting hypotheses into "true" and "false" or declaring "rejection" or "no rejection", without demanding a reasonable interpretation of the results, as has been noted time and again for decades. This binary orthodoxy validates categorical thinking, which results in a very simplistic view of scientific activity that induces researchers not to test theories about the magnitude of effect sizes [18-20].

Despite the weakness and shortcomings of the NHST, they are frequently taught as if they were the key inferential statistical method or the most appropriate, or even the sole unquestioned one. The statistical textbooks, with only some exceptions, do not even mention the NHST controversy. Instead, the myth is spread that NHST is the "natural" final action of scientific inference and the only procedure for testing hypotheses. However, relevant specialists and important regulators of the scientific world advocate avoiding them.

Taking especially into account that NHST does not offer the most important information (i.e. the magnitude of an effect of interest, and the precision of the estimate of the magnitude of that effect), many experts recommend the reporting of point estimates of effect sizes with confidence intervals as the appropriate representation of the inherent uncertainty linked to empirical studies [21-25]. Since 1988, the *International Committee of Medical Journal Editors *(ICMJE, known as the *Vancouver Group*) incorporates the following recommendation to authors of manuscripts submitted to medical journals: "When possible, quantify findings and present them with appropriate indicators of measurement error or uncertainty (such as confidence intervals). Avoid relying solely on statistical hypothesis testing, such as P-values, which fail to convey important information about effect size" [26].

As will be shown, the use of confidence intervals (CI), occasionally accompanied by P-values, is recommended as a more appropriate method for reporting results. Some authors have noted several shortcomings of CI long ago [27]. In spite of the fact that calculating CI could be complicated indeed, and that their interpretation is far from simple [28,29], authors are urged to use them because they provide much more information than the NHST and do not merit most of its criticisms of NHST [30]. While some have proposed different options (for instance, likelihood-based information theoretic methods [31], and the Bayesian inferential paradigm [32]), confidence interval estimation of effect sizes is clearly the most widespread alternative approach.

Although twenty years have passed since the ICMJE began to disseminate such recommendations, systematically ignored by the vast majority of textbooks and hardly incorporated in medical publications [33], it is interesting to examine the extent to which the NHST is used in articles published in medical journals during recent years, in order to identify what is still lacking in the process of eradicating the widespread ceremonial use that is made of statistics in health research [34]. Furthermore, it is enlightening in this context to examine whether these patterns differ between English- and Spanish-speaking worlds and, if so, to see if the changes in paradigms are occurring more slowly in Spanish-language publications. In such a case we would offer various suggestions.

In addition to assessing the adherence to the above cited statistical recommendation proposed by ICMJE relative to the use of P-values, we consider it of particular interest to estimate the extent to which the *significance fallacy *is present, an inertial deficiency that consists of attributing -- explicitly or not -- qualitative importance or practical relevance to the found differences simply because statistical significance was obtained.

Many authors produce misleading statements such as "a significant effect was (or was not) found" when it should be said that "a statistically significant difference was (or was not) found". A detrimental consequence of this equivalence is that some authors believe that finding out whether there is "statistical significance" or not is the aim, so that this term is then mentioned in the conclusions [35]. This means virtually nothing, except that it indicates that the author is letting a computer do the thinking. Since the real research questions are never statistical ones, the answers cannot be statistical either. Accordingly, the conversion of the dichotomous outcome produced by a NHST into a conclusion is another manifestation of the mentioned fallacy.

The general objective of the present study is to evaluate the extent and quality of use of NHST and CI, both in English- and in Spanish-language biomedical publications, between 1995 and 2006 taking into account the *International Committee of Medical Journal Editors *recommendations, with particular focus on accuracy regarding interpretation of statistical significance and the validity of conclusions.

## Methods

We reviewed the original articles from six journals, three in English and three in Spanish, over three disjoint periods sufficiently separated from each other (1995-1996, 2000-2001, 2005-2006) as to properly describe the evolution in prevalence of the target features along the selected periods.

The selection of journals was intended to get representation for each of the following three thematic areas: clinical specialties (*Obstetrics & Gynecology *and *Revista Española de Cardiología)*; Public Health and Epidemiology (*International Journal of Epidemiology *and *Atención Primaria) *and the area of general and internal medicine (*British Medical Journal *and *Medicina Clínica*). Five of the selected journals formally endorsed ICMJE guidelines; the remaining one (*Revista Española de Cardiología*) suggests observing ICMJE demands in relation with specific issues. We attempted to capture journal diversity in the sample by selecting general and specialty journals with different degrees of influence, resulting from their impact factors in 2007, which oscillated between 1.337 (MC) and 9.723 (BMJ). No special reasons guided us to choose these specific journals, but we opted for journals with rather large paid circulations. For instance, the Spanish Cardiology Journal is the one with the largest impact factor among the fourteen Spanish Journals devoted to clinical specialties that have impact factor and *Obstetrics & Gynecology *has an outstanding impact factor among the huge number of journals available for selection.

It was decided to take around 60 papers for each biennium and journal, which means a total of around 1,000 papers. As recently suggested [36,37], this number was not established using a conventional method, but by means of a purposive and pragmatic approach in choosing the maximum sample size that was feasible.

Systematic sampling in phases [38] was used in applying a sampling fraction equal to 60/N, where N is the number of articles, in each of the 18 subgroups defined by crossing the six journals and the three time periods. Table 1 lists the population size and the sample size for each subgroup. While the sample within each subgroup was selected with equal probability, estimates based on other subsets of articles (defined across time periods, areas, or languages) are based on samples with various selection probabilities. Proper weights were used to take into account the stratified nature of the sampling in these cases.

**Table 1:** Sizes of the populations (and the samples) for selected journals and periods.

	Clinical		General Medicine		Public Health and Epidemiology		
**Period**	**G&O**	**REC**	**BMJ**	**MC**	**IJE**	**AP**	**Total**

1995-1996	623 (62)	125 (60)	346 (62)	238 (61)	315 (60)	169 (60)	1816 (365)

2000-2001	600 (60)	146 (60)	519 (62)	196 (61)	286 (60)	145 (61)	1892 (364)

2005-2006	537 (59)	144 (59)	474 (62)	158 (62)	212 (61)	167 (60)	1692 (363)

Total	1760 (181)	415 (179)	1339 (186)	592 (184)	813 (181)	481 (181)	5400 (1092)

G&O: *Obstetrics & Gynecology; *REC: *Revista Española de Cardiología; *BMJ: *British Medical Journal; *MC: *Medicina Clínica; *IJE: *International Journal of Epidemiology; *AP: *Atención Primaria*.

**Figure 1:**
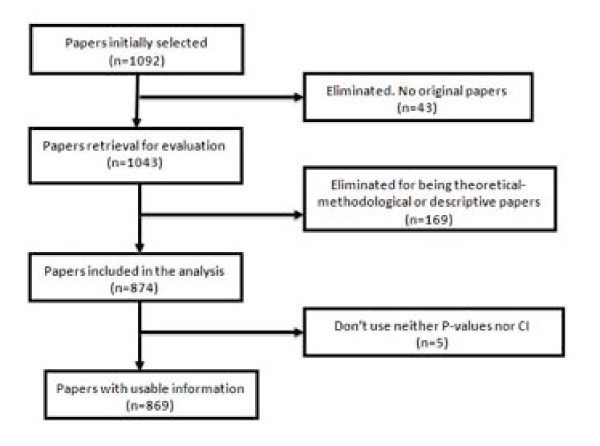
**Flow chart of the selection process for eligible papers**.

Forty-nine of the 1,092 selected papers were eliminated because, although the section of the article in which they were assigned could suggest they were originals, detailed scrutiny revealed that in some cases they were not. The sample, therefore, consisted of 1,043 papers. Each of them was classified into one of three categories: (1) purely *descriptive *papers, those designed to review or characterize the state of affairs as it exists at present, (2) analytical papers, or (3) articles that address theoretical, methodological or conceptual issues. An article was regarded as *analytical *if it seeks to explain the reasons behind a particular occurrence by discovering causal relationships or, even if self-classified as descriptive, it was carried out to assess cause-effect associations among variables. We classify as *theoretical *or *methodological *those articles that do not handle empirical data as such, and focus instead on proposing or assessing research methods. We identified 169 papers as purely descriptive or theoretical, which were therefore excluded from the sample. Figure 1 presents a flow chart showing the process for determining eligibility for inclusion in the sample.

To estimate the adherence to ICMJE recommendations, we considered whether the papers used P-values, confidence intervals, and both simultaneously. By "the use of P-values" we mean that the article contains at least one P-value, explicitly mentioned in the text or at the bottom of a table, or that it reports that an effect was considered as *statistically significant*. It was deemed that an article uses CI if it explicitly contained at least one confidence interval, but not when it only provides information that could allow its computation (usually by presenting both the estimate and the standard error). Probability intervals provided in Bayesian analysis were classified as confidence intervals (although conceptually they are not the same) since what is really of interest here is whether or not the authors quantify the findings and present them with appropriate indicators of the margin of error or uncertainty.

In addition we determined whether the "Results" section of each article attributed the status of "significant" to an effect on the sole basis of the outcome of a NHST (i.e., without clarifying that it is strictly statistical significance). Similarly, we examined whether the term "significant" (applied to a test) was mistakenly used as synonymous with *substantive*, *relevant *or *important*. The use of the term "significant effect" when it is only appropriate as a reference to a "statistically significant difference," can be considered a direct expression of the significance fallacy [39] and, as such, constitutes one way to detect the problem in a specific paper.

We also assessed whether the "Conclusions," which sometimes appear as a separate section in the paper or otherwise in the last paragraphs of the "Discussion" section mentioned statistical significance and, if so, whether any of such mentions were no more than an allusion to results.

To perform these analyses we considered both the abstract and the body of the article. To assess the handling of the significance issue, however, only the body of the manuscript was taken into account.

The information was collected by four trained observers. Every paper was assigned to two reviewers. Disagreements were discussed and, if no agreement was reached, a third reviewer was consulted to break the tie and so moderate the effect of subjectivity in the assessment.

In order to assess the reliability of the criteria used for the evaluation of articles and to effect a convergence of criteria among the reviewers, a pilot study of 20 papers from each of three journals (*Clinical Medicine*, *Primary Care*, and *International Journal of Epidemiology) *was performed. The results of this pilot study were satisfactory. Our results are reported using percentages together with their corresponding confidence intervals. For sampling errors estimations, used to obtain confidence intervals, we weighted the data using the inverse of the probability of selection of each paper, and we took into account the complex nature of the sample design. These analyses were carried out with EPIDAT [40], a specialized computer program that is readily available.

## Results

A total of 1,043 articles were reviewed, of which 874 (84%) were found to be analytic, while the remainders were purely descriptive or of a theoretical and methodological nature. Five of them did not employ either P-values or CI. Consequently, the analysis was made using the remaining 869 articles.

### Use of NHST and confidence intervals

The percentage of articles that use only P-values, without even mentioning confidence intervals, to report their results has declined steadily throughout the period analyzed (Table 2). The percentage decreased from approximately 41% in 1995-1996 to 21% in 2005-2006. However, it does not differ notably among journals of different languages, as shown by the estimates and confidence intervals of the respective percentages. Concerning thematic areas, it is highly surprising that most of the clinical articles ignore the recommendations of ICMJE, while for general and internal medicine papers such a problem is only present in one in five papers, and in the area of Public Health and Epidemiology it occurs only in one out of six. The use of CI alone (without P-values) has increased slightly across the studied periods (from 9% to 13%), but it is five times more prevalent in Public Health and Epidemiology journals than in Clinical ones, where it reached a scanty 3%.

**Table 2:** Prevalence of NHST and CI across periods, languages and research areas.

		Total of papers	P-values and no CI			CI and P-values			CI and no P-values	
			
			n	% (95%CI)		n	% (95%CI)		n	% (95%CI)
Period	1995-1996	285	119	41 (35 to 47)		138	49 (43 to 55)		28	10 (6 to13)
	
	2000-2001	278	101	38 (31 to 44)		150	51 (44 to 58)		27	11 (6 to 15)
	
	2005-2006	306	65	21 (16 to 26)		198	65 (59 to 71)		43	14 (9 to 17)

Language	Spanish	396	156	39 (34 to 43)		211	54 (49 to 59)		29	7 (5 to 10)
	
	English	473	129	32 (28 to 36)		275	55 (51 to 60)		69	12 (10 to 15)

Area	Clinical	300	166	52 (45 to 58)		125	45 (39 to 51)		9	3 (1 to 6)
	
	General Medicine	278	69	22 (17 to 27)		170	61 (55 to 67)		39	17 (12 to 22)
	
	Public Health and Epidemiology	291	50	18 (13 to 23)		191	65 (59 to 71)		50	17 (13 to 22)

CI: Confidence Interval

### Ambivalent handling of the significance

While the percentage of articles referring implicitly or explicitly to significance in an ambiguous or incorrect way - that is, incurring the significance fallacy -- seems to decline steadily, the prevalence of this problem exceeds 69%, even in the most recent period. This percentage was almost the same for articles written in Spanish and in English, but it was notably higher in the Clinical journals (81%) compared to the other journals, where the problem occurs in approximately 7 out of 10 papers (Table 3). The kappa coefficient for measuring agreement between observers concerning the presence of the "significance fallacy" was 0.78 (CI95%: 0.62 to 0.93), which is considered acceptable in the scale of Landis and Koch [41].

**Table 3:** Frequency of occurrence of the significance fallacy across periods, languages and research areas.

Criteria	Categories	Number of papers examined	Frequency of occurrence of the significance fallacy	% (95%CI)
Period	1995-1996	285	224	80 (75 to 85)
	
	2000-2001	278	210	78 (72 to 83)
	
	2005-2006	306	216	70 (64 to 75)

Language	Spanish	396	295	73 (69 to 78)
	
	English	473	355	76 (73 to 80)

Area	Clinical	300	248	81(76 to 86)
	
	General Medicine	278	200	72 (66 to 77)
	
	Public Health and Epidemiology	291	202	71 (66 to 76)

CI: Confidence Interval

### Reference to numerical results or statistical significance in Conclusions

The percentage of papers mentioning a numerical finding as a conclusion is similar in the three periods analyzed (Table 4). Concerning languages, this percentage is nearly twice as large for Spanish journals as for those published in English (approximately 21% versus 12%). And, again, the highest percentage (16%) corresponded to clinical journals.

**Table 4:** Frequency of use of numerical results in conclusions across periods, languages and research areas.

Criteria	Categories	Number of papers examined	Frequency of use of numerical results in conclusions	% (95%CI)
Period	1995-1996	285	44	15 (10 to 19)
	
	2000-2001	278	48	15 (10 to 20)
	
	2005-2006	306	45	12,1 (8 to 16)

Language	Spanish	396	85	21 (17 to 25)
	
	English	473	52	12 (9 to 15)

Area	Clinical	300	58	16 (12 to 21)
	
	General Medicine	278	39	13 (9 to 17)
	
	Public Health and Epidemiology	291	40	12 (8 to 15)

CI: Confidence Interval

A similar pattern is observed, although with less pronounced differences, in references to the outcome of the NHST (significant or not) in the conclusions (Table 5). The percentage of articles that introduce the term in the "Conclusions" does not appreciably differ between articles written in Spanish and in English. Again, the area where this insufficiency is more often present (more than 15% of articles) is the Clinical area.

**Table 5:** Frequency of presence of the term Significance (or statistical significance) in conclusions across periods, languages and research areas.

Criteria	Categories	Number of papers examined	Frequency of presence of significance in conclusions	% (95%CI)
Period	1995-1996	285	35	14 (9 to 19)
	
	2000-2001	278	32	12 (8 to 16)
	
	2005-2006	306	41	14 (9 to 19)

Language	Spanish	396	39	10 (7 to 13)
	
	English	473	69	15 (11 to 18)

Area	Clinical	300	44	16 (11 to 20)
	
	General Medicine	278	30	11 (7 to 15)
	
	Public Health and Epidemiology	291	34	12 (8 to 16)

CI: Confidence Interval

## Discussion

There are some previous studies addressing the degree to which researchers have moved beyond the ritualistic use of NHST to assess their hypotheses. This has been examined for areas such as biology [42], organizational research [43], or psychology [44-47]. However, to our knowledge, no recent research has explored the pattern of use P-values and CI in medical literature and, in any case, no efforts have been made to study this problem in a way that takes into account different languages and specialties.

At first glance it is puzzling that, after decades of questioning and technical warnings, and after twenty years since the inception of ICMJE recommendation to avoid NHST, they continue being applied ritualistically and mindlessly as the dominant doctrine. Not long ago, when researchers did not observe statistically significant effects, they were unlikely to write them up and to report "negative" findings, since they knew there was a high probability that the paper would be rejected. This has changed a bit: editors are more prone to judge *all *findings as potentially eloquent. This is probably the frequent denunciations of the tendency for those papers presenting a significant positive result to receive more favorable publication decisions than equally well-conducted ones that report a negative or null result, the so-called *publication bias *[48-50]. This new openness is consistent with the fact that if the substantive question addressed is really relevant, the answer (whether positive or negative) will also be relevant.

Consequently, even though it was not an aim of our study, we found many examples in which statistical significance was not obtained. However, many of those negative results were reported with a comment of this type: "*The results did not show a significant difference between groups; however, with a larger sample size, this difference would have probably proved to be significant*". The problem with this statement is that it is true; more specifically, it will *always *be true and it is, therefore, sterile. It is not fortuitous that one never encounters the opposite, and equally tautological, statement: "*A significant difference between groups has been detected; however, perhaps with a smaller sample size, this difference would have proved to be not significant"*. Such a double standard is itself an unequivocal sign of the ritual application of NHST.

Although the declining rates of NHST usage show that, gradually, ICMJE and similar recommendations are having a positive impact, most of the articles in the clinical setting still considered NHST as the final arbiter of the research process. Moreover, it appears that the improvement in the situation is mostly formal, and the percentage of articles that fall into the *significance fallacy *is huge.

The contradiction between what has been conceptually recommended and the common practice is sensibly less acute in the area of Epidemiology and Public Health, but the same pattern was evident everywhere in the mechanical way of applying significance tests. Nevertheless, the clinical journals remain the most unmoved by the recommendations.

The ICMJE recommendations are not cosmetic statements but substantial ones, and the vigorous exhortations made by outstanding authorities [51] are not mere intellectual exercises due to ingenious and inopportune methodologists, but rather they are very serious epistemological warnings.

In some cases, the role of CI is not as clearly suitable (e.g. when estimating multiple regression coefficients or because effect sizes are not available for some research designs [43,52]), but when it comes to estimating, for example, an odds ratio or a rates difference, the advantage of using CI instead of P values is very clear, since in such cases it is obvious that the goal is to assess what has been called the "effect size."

The inherent resistance to change old paradigms and practices that have been entrenched for decades is always high. Old habits die hard. The estimates and trends outlined are entirely consistent with Alvan Feinstein's warning 25 years ago: "Because the history of medical research also shows a long tradition of maintaining loyalty to established doctrines long after the doctrines had been discredited, or shown to be valueless, we cannot expect a sudden change in this medical policy merely because it has been denounced by leading connoisseurs of statistics [53]".

It is possible, however, that the nature of the problem has an external explanation: it is likely that some editors prefer to "avoid troubles" with the authors and vice versa, thus resorting to the most conventional procedures. Many junior researchers believe that it is wise to avoid long back-and-forth discussions with reviewers and editors. In general, researchers who want to appear in print and survive in a publish-or-perish environment are motivated by force, fear, and expedience in their use of NHST [54]. Furthermore, it is relatively natural that simple researchers use NHST when they take into account that some theoretical objectors have used this statistical analysis in empirical studies, published after the appearance of their own critiques [55].

For example, *Journal of the American Medical Association *published a bibliometric study [56] discussing the impact of statisticians' co-authorship of medical papers on publication decisions by two major high-impact journals: *British Medical Journal *and *Annals of Internal Medicine*. The data analysis is characterized by methodological orthodoxy. The authors just use chi-square tests without any reference to CI, although the NHST had been repeatedly criticized over the years by two of the authors:

Douglas Altman, an early promoter of confidence intervals as an alternative [57], and Steve Goodman, a critic of NHST from a Bayesian perspective [58]. Individual authors, however, cannot be blamed for broader institutional problems and systemic forces opposed to change.

The present effort is certainly partial in at least two ways: it is limited to only six specific journals and to three biennia. It would be therefore highly desirable to improve it by studying the problem in a more detailed way (especially by reviewing more journals with different profiles), and continuing the review of prevailing patterns and trends.

## Conclusions

Overall, results of our review show some improvements in statistical management of statistical results, but further efforts by scholars and journal editors are clearly required to move the communication toward ICMJE advices, especially in the clinical setting, which seems to be imperative among publications in Spanish.

## Competing interests

The authors declare that they have no competing interests.

## Authors' contributions

LCSA designed the study, wrote the paper and supervised the whole process; PSG coordinated the data extraction and carried out statistical analysis, as well as participated in the editing process; AFS extracted the data and participated in the first stage of statistical analysis; all authors contributed to and revised the final manuscript.

## Pre-publication history

The pre-publication history for this paper can be accessed here:

http://www.biomedcentral.com/1471-2288/10/44/prepub
